# Supplementary material for: Candidate correlates of protection in the HVTN505 HIV-1 vaccine efficacy trial identified by positive-unlabeled learning
Source: PLoS Comput Biol. 2025 Nov 26;21(11):e1013705. doi: 10.1371/journal.pcbi.1013705 (PMC12654939; doi:10.1371/journal.pcbi.1013705)
Supplement: S1 File — S1 Table. Clinical information for the vaccine recipients according to HIV acquisition status and inferred protection status outcomes. S2 Table. Mann-Whitney U statistics for selected immunogenicity features. S1 Fig. Score robustness. PU scores for the unlabeled class across modeling iterations. Symbol indicates mean and error bars indicate standard deviation. Samples are ordered from smallest to largest PU score. S2 Fig. Support for protection status inference validity. PU score for high behavioral risk participants who did not acquire HIV (purple), and those who did (orange). Welch’s test p = 0.047. S3 Fig. Logistic regression to study relationships between immune features and prediction outcome after adjusting for clinical traits as covariates. A. Volcano plot of immune response features under HIV acquisition (left) and inferred (i) protection status (right) outcomes from a logistic regression model. Regression model fit with (top) and without (bottom) adjustment for clinical covariates. The Benjamin-Hochberg procedure was used to adjust p-values from Mann Whitney U test to reduce false discovery rate. B. Plot of odds ratio values for each immunogenicity feature under HIV acquisition (left) and inferred protection (right) class labels. Error bars represent the 95% confidence interval (CI) and are colored according to feature type. Dotted line indicates no association. (PDF) [file pcbi.1013705.s001.pdf]

Supplementary Materials

**Supplementary Table**	
Supplementary Table 1	Clinical information for the vaccine recipients according to HIV acquisition status and inferred protection status outcomes.
Supplementary Table 2	Mann-Whitney U statistics for selected immunogenicity features.
**Supplementary Figure**	
Supplementary Figure 1	Score robustness.
Supplementary Figure 2	Support for protection status inference validity.
Supplementary Figure 3	Logistic regression to study relationships between immune features and prediction outcome after adjusting for clinical traits as covariates.

Variable	Level	Vaccinee	Infected	Uninfected	I: Unprotected	I: Protected
Sample Size (N)	N/A	150	25	125	78	72
Age (SD)	N/A	32.6 (9.2)	30.5 (8.45)	33 (9.34)	31.1(9.12)	34.2 (9.12)
Race (%)	White	90 (60)	15 (60)	75 (0.6)	46 (59.0)	44
	Black	42 (28)	7 (28)	35 (0.28)	20 (25.6)	22
	Hispanic/others	18 (12)	3(12)	15 (0.12)	12 (15.4)	6
Body Mass Index (SD)	N/A	26.3 (4.7)	25.83	26.4 (5.82)	26.5 (4.63)	26.2 (4.90)
BMI Level (%)	Healthy [18.4-25)	68 (45.3)	12 (48)	56 (44.8)	32 (41)	36 (50)
	Overweight [25-29.8)	46 (30.7)	8 (32)	38	26 (33.3)	20 (27.8)
	Obesity [29.8, Inf)	36 (24.0)	5 (20)	31	20 (25.6)	16 (22.2)
Behavioral Risk Score (SD)	N/A	0.457(0.382)	0.618 (0.391)	0.425 (0.374)	0.491 (0.4)	0.421 (0.363)
Behavioral Risk Level (%)	High	38 (25.3)	11(44)	27 (21.6)	24 (30.8)	14 (19.4)
	Medium	62 (41.3)	9(36)	83 (66.4)	29 (37.2)	33 (45.8)
	Low	50 (33.3)	5(20)	15 (12)	25 (32.1)	25 (34.7)

Supplementary Table 1: Clinical information for the vaccine recipients according to HIV acquisition status and inferred protection status outcomes.

	U statistic	
Feature	Acquisition	$_{inf}$Protection
ADCP	1027	1214
IgG3	892	786
FcγRIIa	1159	1662
FcγRIIIa	1075	1307
IgG	1237	2017
IgA	1407	1912

Supplemental Table 2. Mann-Whitney U statistics for selected immunogenicity features

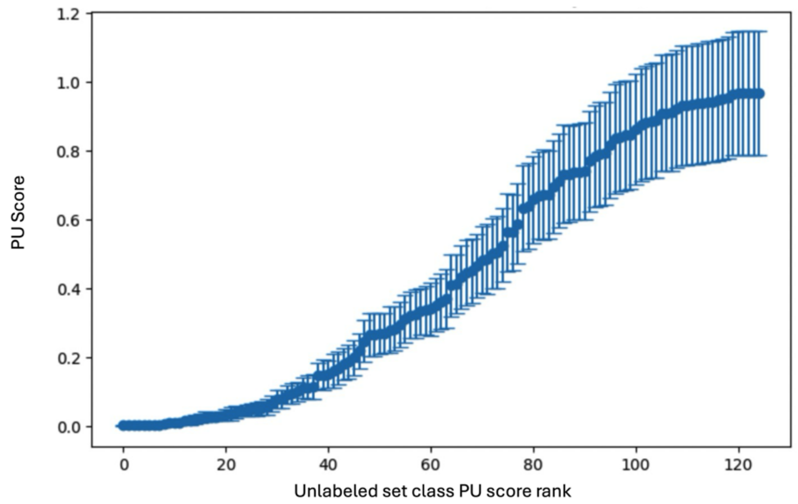

Supplementary Figure 1: Score robustness. PU scores for the unlabeled class across modeling iterations (repeated scoring across different bagging seeds). Symbol indicates mean and error bars indicate standard deviation. Samples are ordered from smallest to largest PU score.

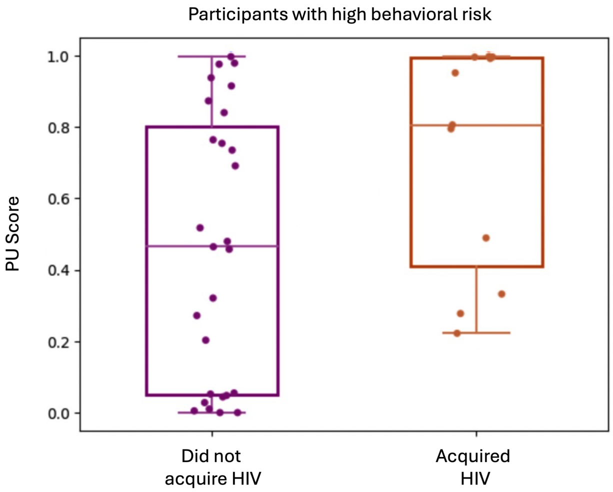

Supplementary Figure 2: Support for protection status inference validity. PU score for high behavioral risk participants who did not acquire HIV (purple), and those who did (orange). Welch's test p=0.047.

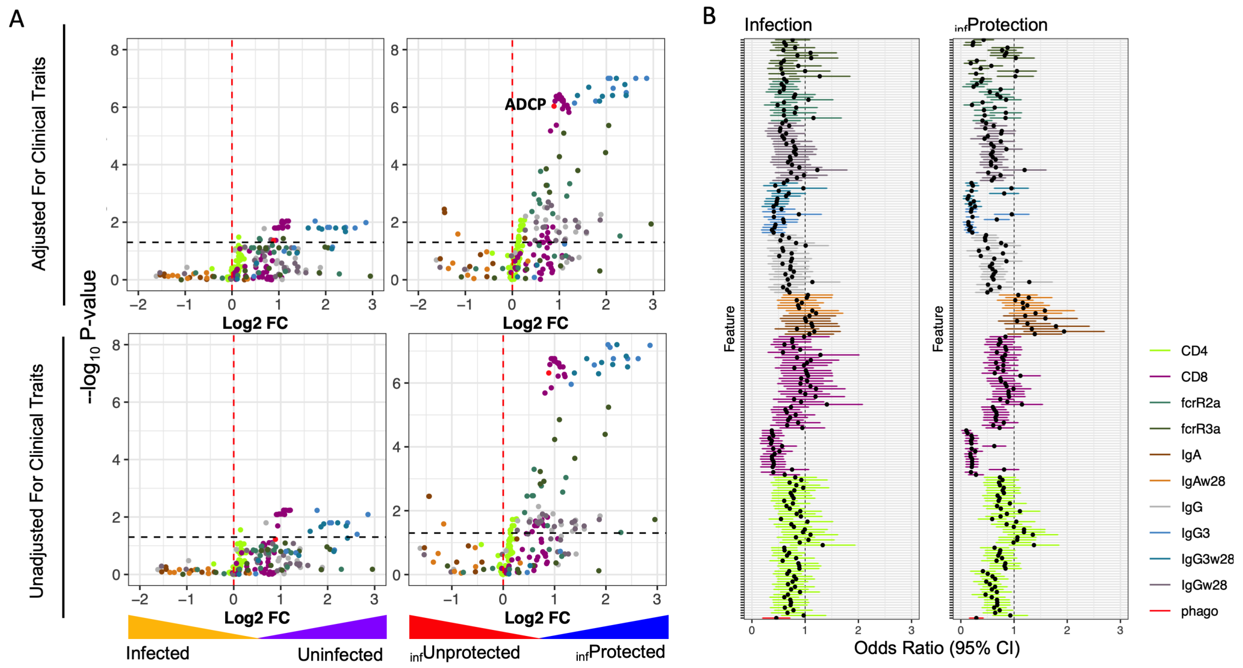

Supplementary Figure 3: Logistic regression to study relationships between immune features and prediction outcome after adjusting for clinical traits as covariates. A. Volcano plot of immune response features under HIV acquisition (left) and inferred (i) protection status (right) outcomes from a logistic regression model. Regression model were fit with (top) and without (bottom) adjustment for clinical covariates. The Benjamin-Hochberg procedure was used to adjust p-values from Mann Whitney U test to reduce false discovery rate. **B**. Plot of odds ratio values for each immunogenicity feature under HIV acquisition (left) and inferred protection (right) class labels. Error bars represent the 95% confidence interval (CI), and are colored according to feature type. Dotted line indicates no association.
