# Supplementary material for: Candidate correlates of protection in the HVTN505 HIV-1 vaccine efficacy trial identified by positive-unlabeled learning
Source: PLoS Comput Biol. 2025 Nov 26;21(11):e1013705. doi: 10.1371/journal.pcbi.1013705 (PMC12654939; doi:10.1371/journal.pcbi.1013705)
Supplement: Attachment — Submitted filename: PCOMPBIOL-D-24-01909_R3viewer 3.pdf [file pcbi.1013705.s002.pdf]

PLOS Computational Biology

Expanded Insights into Correlates of Protection in the HVTN505 HIV-1 Vaccine Efficacy Trial Afforded by Positive-Unlabeled Learning

--Manuscript Draft--

**Manuscript Number:**	PCOMPBIOL-D-24-01909
**Full Title:**	Expanded Insights into Correlates of Protection in the HVTN505 HIV-1 Vaccine Efficacy Trial Afforded by Positive-Unlabeled Learning
**Short Title:**	Correlates of Protection in the HVTN505 HIV-1 Vaccine Efficacy Trial
**Article Type:**	Research Article
**Keywords:**	Phase IIb/III trial; vaccine efficacy; correlates of risk; correlates of protection; HVTN 505; HIV-1 vaccine; positive-unlabeled learning; case-control analysis
**Abstract:**	With a goal of unveiling mechanisms by which vaccines can provide protection against HIV-1 acquisition, several studies have explored correlates of risk of HIV-1 acquisition in HVTN 505, which was a phase IIb trial conducted to assess the safety and efficacy of a DNA plasmid and recombinant adenovirus serotype 5-vectored HIV vaccine regimen among individuals in the United States who were vulnerable to acquiring HIV. While this trial failed to meet its predetermined efficacy criteria, both immunological and virological correlates of reduced risk of acquisition have been reported, suggesting that at least some vaccine recipients were protected from some viruses. In this work, we describe application of a novel Positive-Unlabeled learning-based approach to infer protection status among vaccine recipients without HIV resulting in improved power to detect potential correlates of immunity. Having established the robustness of protection status predictions using a permutation testing strategy, we report increased confidence in previously identified correlates of risk, such as vaccine-elicited anti–HIV-1 Env glycoprotein IgG3 antibodies and antibody-dependent phagocytosis, and the new observation of a strong inverse correlation between vaccine-mediated protection and virus-specific IgA responses. This inference approach offers a new means to use case-control datasets to identify markers of effective immune responses in the context of low vaccine efficacy.
**Additional Information:**	
**Question**	**Response**
**Government Employee**<br><br>Are you or any of the contributing authors an employee of the United States government?<br><br>Manuscripts authored by one or more US Government employees are not copyrighted, but are licensed under a CC0 Public Domain Dedication, which allows unlimited distribution and reuse of the article for any lawful purpose. This is a legal requirement for US Government employees.<br><br>This will be typeset if the manuscript is accepted for publication.	No - No authors are employees of the U.S. government.

Powered by Editorial Manager® and ProduXion Manager® from Aries Systems Corporation

**Financial Disclosure**<br><br>Enter a financial disclosure statement that describes the sources of funding for the work included in this submission. Review the submission guidelines for detailed requirements. View published research articles from *PLOS Computational Biology* for specific examples.<br><br>This statement is required for submission and **will appear in the published article** if the submission is accepted. Please make sure it is accurate.<br><br>**Funded studies**<br>Enter a statement with the following details:<br>• Initials of the authors who received each award<br>• Grant numbers awarded to each author<br>• The full name of each funder<br>• URL of each funder website<br>• Did the sponsors or funders play any role in the study design, data collection and analysis, decision to publish, or preparation of the manuscript?<br><br>Did you receive funding for this work?	Yes
Please add funding details.<br>as follow-up to "**Financial Disclosure**<br><br>Enter a financial disclosure statement that describes the sources of funding for the work included in this submission. Review the submission guidelines for detailed requirements. View published research articles from *PLOS Computational Biology* for specific examples.<br><br>This statement is required for submission and **will appear in the published article** if the submission is accepted. Please make sure it is accurate.	This study was supported in part by NIAID R56AI165448 and P01AI162242 and by the HVTN505 study team NIH 5UM1AI068618.

**Funded studies**<br>Enter a statement with the following details:<br>• Initials of the authors who received each award<br>• Grant numbers awarded to each author<br>• The full name of each funder<br>• URL of each funder website<br>• Did the sponsors or funders play any role in the study design, data collection and analysis, decision to publish, or preparation of the manuscript?<br><br>Did you receive funding for this work?"	
Please select the country of your main research funder (please select carefully as in some cases this is used in fee calculation).<br>as follow-up to "**Financial Disclosure**<br><br>Enter a financial disclosure statement that describes the sources of funding for the work included in this submission. Review the submission guidelines for detailed requirements. View published research articles from *PLOS Computational Biology* for specific examples.<br><br>This statement is required for submission and **will appear in the published article** if the submission is accepted. Please make sure it is accurate.<br><br>**Funded studies**<br>Enter a statement with the following details:<br>• Initials of the authors who received each award<br>• Grant numbers awarded to each author<br>• The full name of each funder<br>• URL of each funder website<br>• Did the sponsors or funders play any role in the study design, data collection and analysis, decision to publish, or preparation of the manuscript?<br><br>Did you receive funding for this work?"	UNITED STATES - US
**Competing Interests**	The authors have declared that no competing interests exist.

On behalf of all authors, disclose any competing interests that could be perceived to bias this work.<br><br>This statement will be typeset if the manuscript is accepted for publication.<br><br>Review the instructions link below and PLOS Computational Biology's competing interests policy to determine what information must be disclosed at submission.	
**Data and Code Availability**<br><br>From the time of publication, Authors are required to make fully available and without restriction all data and computational code underlying their findings. Please see our PLOS Data Policy page for detailed policy information, and our Code Sharing page for specific information on code sharing.<br><br>A **Data Availability Statement**, detailing where the data (and code, if applicable) can be accessed, is required at first submission. Insert your Data Availability Statement in the box below. The statement you provide **will be published in the article**, if accepted.<br><br>Please see the Data Reporting section of our submission guidelines for instructions on what you need to include in your Data Availability Statement.<br><br>If the data and code are all contained in your submission files, please state: *All relevant data are within the manuscript and its Supporting Information files.*<br><br>**PLOS allows rare exemptions to address legal and ethical concerns.** If you have legal or ethical restrictions, please detail these in your Data Availability Statement below for the Journal team to consider.	The datasets used in this analysis are available at https://atlas.scharp.org/project/HVTN%20Public%20Data/HVTN%20505/begin.view.<br><br>Code will be made available upon publication.

Powered by Editorial Manager® and ProduXion Manager® from Aries Systems Corporation

Manuscript

Click here to access/download;Manuscript;Manuscript Xu HVTN505_110524.docx

Expanded Insights into Correlates of Protection in the HVTN505 HIV-1 Vaccine Efficacy Trial Afforded by Positive-Unlabeled Learning

Shiwei Xu[1], Aaron Hudson[2], Holly E. Janes[2], Georgia D. Tomaras[3], Margaret E. Ackerman[14,5]*

[1]Quantitative Biomedical Sciences Program, Dartmouth College, Hanover, NH USA

[2]Biostatistics, Bioinformatics and Epidemiology Program, Vaccine and Infectious Disease Division, Fred Hutch Cancer Center, Seattle, WA

[3]Department of Surgery, Center for Human Systems Immunology, Duke University, Durham, SC, USA

[4]Department of Microbiology and Immunology, Geisel School of Medicine at Dartmouth, Dartmouth College, Hanover, NH, USA

[5]Thayer School of Engineering, Dartmouth College, Hanover, NH, USA

*Corresponding author

Margaret E. Ackerman
14 Engineering Dr.
Hanover, NH 03755
margaret.e.ackerman@dartmouth.edu
603 646 9922

Abstract

With a goal of unveiling mechanisms by which vaccines can provide protection against HIV-1 acquisition, several studies have explored correlates of risk of HIV-1 acquisition in HVTN 505, which was a phase IIb trial conducted to assess the safety and efficacy of a DNA plasmid and recombinant adenovirus serotype 5-vectored HIV vaccine regimen among individuals in the United States who were vulnerable to acquiring HIV. While this trial failed to meet its predetermined efficacy criteria, both immunological and virological correlates of reduced risk of acquisition have been reported, suggesting that at least some vaccine recipients were protected from some viruses. In this work, we describe application of a novel Positive-Unlabeled learning-based approach to infer protection status among vaccine recipients without HIV resulting in improved power to detect potential correlates of immunity. Having established the robustness of protection status predictions using a permutation testing strategy, we report increased confidence in previously identified correlates of risk, such as vaccine-elicited anti–HIV-1 Env glycoprotein IgG3 antibodies and antibody-dependent phagocytosis, and the new observation of a strong inverse correlation between vaccine-mediated protection and virus-specific IgA responses. This inference approach offers a new means to use case-control datasets to identify markers of effective immune responses in the context of low vaccine efficacy.

Keywords

Phase IIb/III trial, vaccine efficacy, correlates of risk, correlates of protection, HVTN 505, HIV-1 vaccine, positive-unlabeled learning, case-control analysis

Introduction

One challenge to development of an efficacious HIV-1 vaccine is a lack of clear and reproducible immune correlates of protection, measures or traits that definitively relate to protection from HIV acquisition, which serve to provide understanding of the required immune response to prevent HIV acquisition, and can be reliable biomarkers to predict vaccine efficacy in humans [1]. Among vaccine efficacy trials conducted to date, RV144 (NCT00223080) is the only clinical trial to meet predetermined endpoint efficacy criteria, demonstrating a moderate efficacy of 31.2% (95% confidence interval, 1-51%) at three years [2]. In contrast, in 2013, HIV Vaccine Trial Network (HVTN) 505 (NCT00865566), a multi-center, placebo-controlled preventive HIV-1 vaccine efficacy trial conducted in the United States with 2504 participants enrolled was terminated for vaccine efficacy futility [3]. This study specifically assessed the safety and efficacy of a DNA prime and recombinant adenovirus serotype 5 (rAd5) boost vaccine regimen (DNA/rAd5) among cisgender men and transgender women who had sex with men. Despite lack of overall efficacy, sequence analysis of infecting strains conducted by deCamp et al. [4] showed greater distance between envelope glycoprotein (Env) sequences and the subtype B vaccine insert among vaccine- compared to placebo-recipients, suggesting that vaccination influenced susceptibility to at least some circulating strains.

Consistent with this hypothesis, multiple correlates of risk (CoR), measures or traits that relate to the acquisition status of trial participants, have been reported from this trial using a number of analytical approaches. With Cox proportional hazard models, Janes et al. [5] identified associations between Env-specific CD8+ immune responses and HIV-1 acquisition risk. Neidich et al. [6] performed a CoR analysis with both logistic regression and an ensemble classifier and reported several humoral correlates, including Env-specific IgG3, Env-specific antibody binding to FcγRIIa, and antibody phagocytic activity. While further support of the biological validity of these factors could be found in associations between Env-specific antibody binding to FcγRIIa and viral loads among participants who did acquire HIV, concerns remain: early study termination resulted in a small number of cases, limiting statistical power, and immune markers tended to exhibit only low to moderate statistical significance between acquisition cases and controls without HIV.

Similarly, the Antibody Mediated Prevention (AMP) passive vaccination trials, which tested the ability of the broadly neutralizing antibody VRC01 to prevent HIV acquisition, also failed to meet its primary endpoint criteria of reducing HIV acquisition [7]. However, differences in the sequences [8] and sensitivity [7] of infecting viruses to neutralization by VRC01 between active and placebo study arms have been interpreted as proof of concept for the ability of broadly neutralizing antibodies to provide protection from acquisition of sensitive strains. Indeed, efficacy for reduced likelihood of acquisition by the subset of circulating viruses that could be effectively neutralized by VRC01 was high (~75%). Further, the likelihood of acquisition was inversely proportional to the estimated level of antibody and neutralization titers present at the projected time of acquisition [9, 10]. Lastly, evidence of efficacy can also be found in the observation of reduced viral load among breakthrough cases [11]. Thus, while some might consider analysis of CoR in a trial with such an “ineffective” intervention to be simply misguided, much was learned from such analysis

in the AMP trials. Differences in the real and estimated power of different studies to capture overall efficacy and these more nuanced effects can be used to appropriately soften interpretation of such “failures”. Indeed, these studies provide clear evidence as to the value of case-control analysis even when formal overall endpoint efficacy criteria have not been met.

Although CoR analysis is a mainstay of vaccine research, traditional case-control analytical approaches using acquisition status groups present limited power to unveil correlates of protection (CoP) when acquisition rates are low and exposure status (and therefore actual protection status) cannot be determined. In such cases the class that did not experience acquisition is only a modest proxy for the protected class, and CoR analysis may fail to identify true CoP. While over-representation of controls is a commonly used means to improve upon the ability to detect CoR, alternatives to improving power by biasing sampling between acquisition cases and controls may offer the potential to discover correlates missed by this approach.

In previous work, we have established proof of concept for the potential of semi-supervised machine learning (ML) approaches to contribute to discovery of novel candidate correlates of protection by using acquisition outcome and immunogenicity data to make inferences as to the protection status of study participants that did not acquire HIV [12-14]**.** Specifically, these ML methods aim to distinguish groups with and without HIV in the absence of definitive examples of the group that is protected from acquisition. In this application, positive and negative groups reflect unprotected and protected classes, respectively. Definitive class labels are only available for a subset of the unprotected group, those who were exposed to and acquired HIV. The lack of definitively labeled negative group examples, that is, demonstrably protected participants, points to the potential utility of Positive-Unlabeled (PU) inference methods. These methods have been previously explored to address this challenge using both simplified [12] and more complex synthetic immunogenicity data [13]. Coupled to permutation testing as a means to establish inference robustness [14], these approaches have demonstrated substantial improvements in the ability to discover CoP in PU learning simulation scenarios generated from real-world immunogenicity profiles in which ground truth protection status has not been considered in making class inferences but has been available for validation. To this end, the success of PU learning-based modeling approaches to reveal true CoP that are missed both when acquisition status rather than inferred protection status groups are compared, as well as when multivariate modeling of acquisition status is performed [13], demonstrates the potential of employing this advanced ML method, despite its inherent bias, to improve our understanding of vaccine-induced protection by reliably identifying the study participants with immunogenicity profiles most similar to those who have acquired HIV.

In this work, we intended to provide additional insight into the immune markers that may have contributed to the protection of some vaccinated participants in HVTN 505 by employing a reproducible PU learning-based pipeline for candidate Correlate of Protection (CoP) identification with an integrated null-hypothesis comparison method. HIV acquisition case-control immunogenicity data was used to predict and classify the protection status of vaccinated participants. To the best of our knowledge, this is the first work

to use real-world human trial case-control immunogenicity data to infer participants' protection levels with PU learning. CoR previously identified by analysis of infection status exhibited increased statistical significance when analyzed based on inferred protection status, supporting the relevance of these CoR as potential CoP. Furthermore, this approach supported identification of additional features as candidate CoP. These discoveries included CoR observed previously in the RV144 trial, demonstrating the potential of PU learning to improve understanding of HIV vaccine-mediated protection and identify immune markers to aid the design and development of an efficacious HIV vaccine.

Results

Protection status inferences

Humoral and T cell immunogenicity data previously reported for 25 participants who acquired HIV after week 28 (cases) and 125 participants who did not acquire HIV over all follow-up (controls) from among vaccine recipients in the HVTN505 trial (**Fig.1A**) were analyzed [3, 6, 15] . The sparse number of vaccine recipients who acquired HIV have been definitively exposed to the virus and are also demonstrably unprotected by the vaccine. In contrast, given the assumptions that the vaccine did have some, albeit small, protective effect and that exposure rates are low, the vaccine recipient group who did not acquire HIV is presumed to be a mixture comprising a minority of protected (if exposed, would not have acquired HIV) and a majority of unprotected (if exposed, would have acquired HIV) individuals. The PU learning pipeline employed seeks to infer the protection status of these unlabeled vaccine recipients without HIV (hereafter "unlabeled") based on defining the degree of similarity in their response profiles to the vaccine recipients with HIV, and then to use these newly inferred protection status class labels to identify candidate CoP (**Fig.1B**).

We sought to employ a state-of-the-art PU learning method, termed PU Bootstrapped Aggregation, or Bagging, to predict the control group participants' level of protection based on the degree of similarity of their immunogenicity profiles as compared to those observed among those who acquired HIV. By aggregating predictions from numerous classifiers built with positive class samples and bootstrapped unlabeled class samples, we computed a protection score for each unlabeled control group participant by averaging across their "out-of-bag" (OOB) predictions. A binary protection status classification boundary was then defined with or without knowledge as to the proportion of "protected" participants expected within the cohort using the rank of this score such as provided by the calculation of vaccine efficacy. Confidence in the robustness of these scores and their associated class inferences was evaluated using permutation tests [14]. Lastly, inferred protection status classes that met robustness testing criteria were then used to identify candidate CoPs. With the goal of CoP discovery in mind, and especially in cases where the same immunogenicity data is used to infer protection status as well as be evaluated as potential CoP, exceeding the performance baseline set by permutation testing is key to combating type I errors and over-optimism in identification of potential CoP expected to result from circular analysis.

Dimensionality reduction methods were used to visualize relationships between immunogenicity data and HIV acquisition status outcomes in an unsupervised fashion (**Fig.2A**). Visual inspection of serostatus labels following Principal Component Analysis (PCA) and t-distributed Stochastic Neighbor Embedding (T-SNE) showed some evidence of clustering among the positive case samples within the larger space observed for the control samples. This pattern suggested that semi-supervised approaches could have the potential to differentiate among unlabeled samples that were more or less similar to the known positive class.

Using a previously described and validated PU Bagging approach [16] , each sample was assigned a score reflecting its likelihood of classification as an unprotected (positive) sample (**Fig.2B**). Reassuringly, visual inspection of PU Bagging scores appeared concordant with expectations based on HIV acquisition status labels, however, a rigorous means to assess the robustness of these class inferences was needed. To this end, given the absence of ground truth protection status labels to validate PU Bagging score-based predictions, we employed a positive set evaluation method to serve as a confidence test for the robustness of prediction results. We used permutation testing to determine whether the degree of concordance obtained between scores of known positive samples yielded for the participants who acquired HIV exceeded scores that observed when HIV acquisition status labels were permuted and permuted "known positive" class samples were drawn at random. While such a test cannot establish that class predictions are biologically correct, it can effectively evaluate whether they are statistically different than would be expected at random; that is, it allows us to establish confidence in whether the case group does or does not have a distinct immune response from members of the control group drawn by chance.

We first compared the overall positive set PU Bagging scores under true acquisition outcome labels (actual) and those observed across multiple different permuted acquisition labels following label scrambling (permuted). For both repeated ten-fold and leave-one-out cross-validation runs, both explicit positive recall (EPR) and Mean Bagging Score (MBS) values for the actual positive samples typically exceeded those of permuted positive samples, providing evidence of a biological difference in immunogenicity profiles observed in vaccine-recipients who went on to acquire HIV during the trial's follow up period as compared to those who did not (**Fig.2C**). Consistent with previous work [13], for the true positive class, MBS values were more tightly distributed across replicates than were EPR scores under a 30-time repeated k-fold "spy positive" evaluation [17]. Variance in the distribution of these scores after label permutation was considerably greater. Statistically significant differences between actual and permuted HIV acquisition status class labels were observed for both positive set prediction evaluation metrics across various choices of K (**Fig.2D**). While two-sample independent T tests showed the expected inflation of significance associated with an increasing number of permutations, z-score p values were less susceptible to this artifact, and effect size assessed using Cliff's Delta indicated a medium to large difference between actual and permuted positive class scores, providing confidence in the potential biological relevance of protection status inferences based on reliable differentiation of the actual true positives (**Fig.2D**).

While PU Bagging scores provide a means to compare the relative differences in protection inference confidence, they don't provide an explicit boundary between classes. For cases in which the proportion of protected participants among all participants who did not acquire HIV are known or may be reasonably estimated, such as by overall vaccine accuracy, a percentile cutoff based on PU bagging score rank can be used to set the class threshold. Visualization of protection status class labels assigned based on different degrees of efficacy, or to a score set to 0.5, demonstrate how binary class assignments relate to the lead PCs in this study (**Fig.2E).**

Additionally, an exploratory analysis was conducted to assess the correlation between clinical traits and acquisition outcome and the potential confounding effect between covariates among vaccinated participants in the studied cohort **(Supplementary Table 1).** Consistent with what was observed in the entire cohort, an association was identified between behavioral vulnerability (OR = 4.32, p = 0.0216) and acquisition outcome among cases and controls, adjusted for other covariates (BMI, Race, Age) [3]. Intriguingly, a significant association between behavioral vulnerability and inferred protection status classified by the PU Bagging pipeline was not observed (OR = 1.76, p = 0.217) as either a univariate factor or in a multivariate logistic regression model. This result provides evidence that the immunogenicity measurements possess the potential to forecast participants' expected protection level regardless of behavioral vulnerability factors that are correlated to the likelihood of HIV acquisition.

Identification of candidate CoP

We next trained a Random Forest (RF) classifier, a decision tree-based machine learning model, to predict membership in inferred protection (I: Protection) and infection status classes based on immunogenicity data. Stratified five-fold cross-validation was used to fine-tune the hyperparameters of the RF classifier and estimate the generalizability of the established model on unseen data [18]. Classification accuracy, as defined by area under the Receiver Operator Characteristic curve (ROC-AUC), was considerably greater for predictions of inferred protection status (Mean AUC: 0.94; SD: 0.03)) as compared to HIV acquisition status labels (Mean AUC: 0.66; SD: 0.04) (**Fig.3A**), suggesting that the model has learned patterns and dependencies in the data that are relevant to these class inferences, as is expected from its reliance on this data in training. Given that this circularity will drive over-optimism in classification performance, this high degree of accuracy should not be interpreted as validating the protection status predictions, but rather as simply a basis for confidence in the relevance of features used to model inferred protection status.

Given this (expected) high degree of accuracy, we next investigated the immunogenicity response features important to these classifications. As compared to prediction of acquisition status, which relied largely on both CD4 and CD8 T cell response attributes as well as IgG response magnitude, and to a lesser extent on virus-specific IgG3 levels, IgG3 features, supported by CD8 T cell features, and additionally included IgA and antibody-dependent phagocytosis (ADCP) measurements contributed to classification of inferred protection status (**Fig.3B)**. In order to evaluate the robustness of these contributions to predicting

protection status and to directly compare with contributions to acquisition status, we repeated modeling 100 times. Importance scores for both features were significantly greater under the label of inferred protection as compared to that under acquisition outcome (**Fig.3C**), consolidating the association between Env gp140 specific IgA and ADCP responses and vaccine-elicited immune protection as inferred by PU learning.

The robustness of these models was evaluated across 30 repeated cross-validation runs with area under the receive-operator curve (AUC) and balanced accuracies reported as outcome metrics. Whereas the actually inferred but not permuted protection status class labels could be reliably predicted, predictions of actual acquisition status were generally not distinct from permuted acquisition status labels (**Fig.3D**). These results emphasize the difficulty inherent to modeling acquisition status in studies where both vaccine efficacy and pathogen exposure rate is low, and results in significant overlap between the immunogenicity profiles of many participants without HIV with those that were exposed and acquired HIV, and natural confusion between them.

As an alternative to modeling acquisition and inferred protection status classes using combinations of immunogenicity response features, we next evaluated associations between each feature and class assignments by Mann-Whitney U test, a non-parametric bivariate method that determines whether a statistically significant difference is observed between different groups of samples. In general, each of the roughly two dozen individual features that were significantly distinct between acquisition status groups exhibited the same directionality and greater confidence for inferred protection status groups (**Fig.3E**). Beyond Env-specific IgG3 and CD8+ T cellular responses, which showed high importance and significance in both multivariate modeling and bivariate statistical tests for both acquisition and inferred protection classes, we observed increased univariate statistical significance in FcγRIIa- and FcγRIIIa-binding of Env-specific antibodies under the inferred protection as compared to HIV acquisition status class labels (**Fig.3E-F**). Consistent with the increased importance of ADCP and Env gp140–specific IgA responses observed in multivariate models, bivariate statistical significance was also seen in the Mann-Whitney U test (**Fig.3D**). Whereas ADCP activity was roughly two-fold higher among participants inferred to be protected, Env-specific IgA was roughly two-fold lower (**Fig.3F**). In total, roughly twice as many additional features exceeded the nominal significance threshold. Similar results were observed when adjusting for clinical traits (**Supplementary Fig.1**).

A subset of features that were either previously reported as humoral CoR or newly identified here as inferred CoP are shown for the ConS gp140 specificity for individual participants based on their acquisition or inferred protection status (**Fig.4A**). As examples, differences in the relationships between classes for ConS gp140-specific IgG and IgA for were pronounced (**Fig.4B**): whereas a difference was not observed between acquisition status classes ($p = 0.1$ and 0.4 for IgG and IgA, respectively), there was high confidence in a group difference based on inferred protection status ($p = 0.0029$ and 0.00033). Consistent with the unrelated prior RV144 HIV-1 vaccine efficacy study [19, 20], while IgG responses were elevated among participants inferred to have been protected, the opposite directionality was seen for IgA.

Expanding on this latter observation, given that IgA measurements in this study were bimodal, with a large number of values around zero, they were also treated as a categorical factor including two levels, IgA negative (log-transformed value = 0) and IgA positive (log-transformed value > 0). A logistic regression model was employed to determine whether an association was observed between Env gp140-specific IgA+ and IgA- responders and inferred protection class. Here, after adjusting for clinical traits (including age, BMI, behavioral vulnerability, and race), HIV-1 Env Con S gp140 IgA binding MFI were identified to be inversely correlated to inferred vaccine-mediated protection as both a continuous (OR = 1.17, p = 0.0012) as well as a categorical variable (OR = 2.78, p = 0.0035) (**Supplementary Fig.1**). In contrast to inferred protection status, no significant association was observed with acquisition outcome, as previously reported [6]. Overall, this analysis demonstrates that PU learning-based inferences of protection status both strengthened confidence in previously reported CoR as well as identified novel candidate CoPs.

Discussion

HIV remains a significant public health concern, and efforts to develop a safe and effective vaccine against the virus continue despite setbacks. Vaccine concepts derived from strategies to induce protective immunity in association with T cell responses and neutralizing antibodies, as well as antibodies with protective effector functions modeled from either nonhuman primate protection studies or to recapitulate the result of the RV144 trial, have all failed to meet predefined efficacy criteria [21-24]. While promising efforts are underway to elicit MHC-E-restricted and networked T cell epitope responses, and to guide the maturation of neutralizing antibody responses [25-27], we might reasonably anticipate that these strategies will also prove only partially protective, and that methods to improve power to discover candidate CoP will hold value. To this end, while broadly neutralizing monoclonal antibodies provide robust protection against experimental viral challenges in nonhuman primates [28-30], their limits in the face of circulating viral diversity in humans were elucidated by the AMP trials, which also failed to demonstrate overall efficacy despite reducing the likelihood of acquisition of sensitive strains [7]. All this history points to the significant challenges ahead, and suggests the value of maximizing insights afforded by each human HIV-1 vaccine efficacy trial.

In previous work, we applied a PU learning-based analysis pipeline on multiple synthetic and real-world vaccine immunogenicity data sets in which protection status was known but partially blinded in advance of analysis as a means to stress test the approach in cases where predictions could be validated [13]. These analyses also directly compared the performance of fully supervised ML-based models to predict ground truth protection status after training to classify samples by acquisition status with semi-supervised models inferring protection status. In these comparisons, and bounded by the control for type I error provided by permutation testing, PU learning yielded better accuracy in correctly identifying protected individuals. In turn, as compared to those discovered through classification of acquisition status, these results also demonstrated identification of true CoP by PU learning [13].

For HVTN 505, univariate analysis, classical logistic regression, and ML-based modeling identified a set of immunogenicity features related to acquisition outcomes. Confidence in relationships between these features and outcomes was increased for inferred protection status classes; previously reported CoR, including Env-specific IgG3, ADCP, and Fcγ receptor binding of HIV-specific antibodies, which were associated with acquisition status [6], demonstrated more confident associations with inferred protection status. Additionally, this study highlighted that Env-specific IgA, which was not identified as correlated to HIV acquisition in HVTN 505 with case-control analysis based on acquisition outcome, contributed to multivariate models and exhibited statistical significance based on analysis of inferred protection status classes. Two of these features (Env gp140–specific IgA and ADCP), which were not observed among top features in models of acquisition status, are intriguing as there is support for their relevance to acquisition outcomes for the RV144 trial [19, 31-35] and in preclinical studies [36-38] that preceded the Mosaico (HVTN 706/HPX3002) and Imbokodo (HVTN 705/HPX2008) trials.

These results support our hypothesis that PU learning could "recover" markers of protection that are not identified by univariate, multivariate, or ML-based analysis of infection status—yielding the potential to contribute much needed insights about vaccine-mediated protection from HIV acquisition. While different experiments would be required to provide direct validation of either the inferred protection classes or the mechanistic relevance of novel correlates, these observations are supported by prior results as noted above. Nonetheless, validation of protection predictions in real-world data remain a limitation of this study due to the absolute absence of negative class examples representing protected participants in this clinical trial. Given this limitation, several methods were employed to test whether the classification outcomes may be concordant with the "ground truth" protection status. We first evaluated the positive-unlabeled learning method only with positive samples and compared the overall score obtained by this positive set with results under PU label permutation to control for type I errors in inferring protection status. Significant increases in positive class scores were observed under the actual HIV acquisition status labels compared to those observed following permutation, suggesting that immunogenicity profiles for participants who acquired HIV (true positives) were sufficiently distinct from at least some of the unlabeled samples as to have the potential to predict protection status among the participants without HIV. Furthermore, we studied the correlation between multiple previously described clinical traits to the classification outcome, including BMI, age, ethnicity, and behavioral vulnerability score. Promisingly, the significant correlation between behavioral vulnerability and acquisition outcomes was not observed for the inferred protection status classes, suggesting that while the likelihood of acquisition is related to behavior, protection status may not be.

On the other hand, inferred protection status is expected to be influenced by the set of feature inputs used. While feature sets that do not have reliable relationships with outcomes could be identified by permutation testing and used to reject overoptimistic protection inferences, it is likely that robust inferences supported by different data sets would nonetheless identify a non-identical set of samples or confidence values. To address this limitation, other data streams, such as transcriptomic profiles [39], could be employed for modeling. Hypothetically, concordance between protection status predicted by different sets

and types of biological measurements would be observed among the same cohort of participants for accurate predictions, and use of one data stream for protection inferences and the other for correlate discovery would be a “data thinning” approach that would more rigorously avoid the signal leakage inherent in the approach used here. Holding out features instead of samples would similarly permit separation of inferences of protection status from identification of candidate CoP, though immunogenicity data can exhibit a high degree of correlation. Each of these approaches is expected to decrease the rate of false positive observations, which despite permutation testing results that support the non-randomness of class inferences, can persist at the level of individual candidate CoP [13]. Because false positives can nonetheless be expected at some rate, caution needs to be paid when interpreting both the classification label inferred by the PU learning method as well as candidate CoP; whether an individual has truly been protected by the vaccine remains unknown.

Overall, while the combination of machine learning approaches and advances in experimental approaches to generate large-scale, high-dimensional genetic and molecular profiles have driven an unprecedented improvement in our understanding of the immune system, knowledge gaps remain. Case-control sample evaluation of other HIV vaccine efficacy studies is ongoing [40, 41]. Here, we demonstrate the potential of PU learning to refine our understanding of correlates of protection and gain better insight into the complex humoral response features that may contribute to vaccine-mediated protection.

Methods and Materials

Datasets

This study utilized case-control immunogenicity and clinical information from 189 participants in the HVTN 505 clinical trial: 150 received a recombinant DNA plasmid vaccine injection followed by a recombinant adenoviral serotype vector vaccine injection, and 39 received placebo injections. HIV acquisition was assessed between Months 7 and 24. Vaccine recipients (N=150) were labeled as “Positive” if HIV was diagnosed (N = 25) during the follow-up period, and unlabeled (N=125) if they were not diagnosed (**Fig.1A**). These cases and controls were matched on a set of criteria as specified in Janes et al., [15].

Immunogenicity Features

As described by Neidich et al., Antibody-Dependent Cellular Phagocytosis (ADCP) [42], log-transformed multiplex Binding Antibody Multiplex Assay (BAMA) [43] and Fc Array assay [44, 45] fluorescence intensities, and CD4+ & CD8+ T cellular responses [15] were assayed at 7 months after the first dose of vaccine [6]. To decrease false discoveries, a Wilcoxon test with Benjamini-Hochberg Procedure was performed on each immune feature to compare responses in placebo and vaccinated participants. Immunogenicity features for which $p > 0.05$ were excluded from further analysis based on lack of a vaccine-associated response.

Clinical Information

HVTN 505 study participants were restricted to cisgender men and transgender women who had sex with men. As described by Hammer et al. [3], the age, body mass index, ethnic information, and behavioral vulnerability scores were identified based on a weighted average of two variables from the ACASI questionnaires that were collected at baseline for each participant. Baseline characteristics of sample donors based on HIV acquisition status and inferred protection status groups are presented in **Supplemental Table 1**.

Positive Unlabeled Learning

Positive-unlabeled bootstrapped aggregation (Bagging) algorithms were first adapted to score the probability of the unlabeled samples being classified as “unprotected”. We bootstrapped samples from the unlabeled class with matched size to known positive samples and built a classifier to classify the remaining unlabeled samples. By iteratively bootstrapping different sets of unlabeled samples and predicting the rest of the unlabeled samples with established classifiers, the probability score of each unlabeled sample to be classified as “unprotected” (hidden positive) was calculated as the sum of its prediction when “out-of-bag” (OOB) divided by the number of iterations for which the sample was OOB. In this work, the number of total bootstrapped iterations was set to 200. A support vector machines (SVM) classifier with radial basis function (RBF) kernel, which demonstrated memory efficiency and effectiveness in high dimensional datasets, was used for the PU Bagging approach. To reduce the variance from a single-time prediction, the final PU bagging score for each unlabeled sample was averaged across 30 repetitions.

Positive Set Evaluation

Because samples from definitively protected participants are not available to validate negative class predictions, confidence in predictions can only be assessed using the class of participants who acquired HIV. We therefore implemented testing to score the positive examples. Analogous to cross-validation, the samples that were labeled positive were randomly allocated into 5 folds. Each fold was rotated as “spy positive” samples and mixed with unlabeled samples. On each iteration, PU bagging SVM (RBF) was performed between the (k-1) folds of positive samples and unlabeled samples plus “spy positive” samples. Two scoring methods were utilized to assess the positive set using the PU bagging score of each positive sample as “spy positive”. By treating samples with PU bagging scores ≥0.5 as positive, explicit positive recall (EPR) was adapted to score the positive set according to the proportion of replicates in which positive samples were correctly predicted.

$$Explicit\ Positive\ Recall(EPR) = \frac{N_{PredictedPositive}}{N_{Positive}}$$

$$Mean\ Bagging\ Score\ (MBS) = \frac{\sum\ scores\ from\ PU\ Bagging}{N_{Positive}}$$

An additional scoring method, termed Mean Bagging Score (MBS), was calculated by averaging the PU bagging score of all positive samples. In this work, 30 different fold splits of the positive set were performed under the actual label of infection.

Permutation Comparison

To establish a baseline comparison of the positive class scores (EPR and MBS) obtained, a permutation test was leveraged. In permutation comparisons, the class label of HIV acquisition was randomly shuffled multiple times, and the scores of the resulting permuted infected (P') set were calculated. In this work, the number of permutations was set to 30 or 100. Three statistical methods were employed to compare scores. Welch's T-test and Cliff's Delta statistics were applied to compare the scores for actual HIV serostatus labels as well as for permuted acquisition label. P-values of group z-scores were calculated between the sample mean of scores from actual labels and the distribution of scores from permuted labels. The z score was calculated as:

$$z = \frac{\mu - \mu 0}{\sigma}$$

Where μ denotes the mean score of positive sets based on 30 repetitions, while μ0 represents the mean score obtained by repeatedly permuting the labels and σ refers to the standard deviation of the score distribution within the permutation group, calculated using n-1 degrees of freedom. In this analysis, an upper-tailed test was conducted, assuming the hypothesis that $\mu > \mu 0$.

Inferring Label of Varied Protection Level

Class scores were used to assign binary protection status class labels. Unlabeled samples were classified according to multiple cutoffs based on PU bagging score rank and the hypothetical level of protection (10%, 30%, 50% hypothesized protected). In the absence of information regarding the expected level of protection, binary protection status labels were inferred by aggregating the votes of the predictions from three previously described labeling strategies [13]: (1) Inferring binary class labels according to bagging score cutoff at 0.5, (2) Using 3-fold cross-validation to train a fine-tuned SVM classifier with known positive and matched number samples with lowest score rank as "reliable negative (RN)" samples, which was used to classify the rest of the unlabeled samples, (3) Training a biased classifier with known positive and RN (N_{RN}=25), then, iteratively expanding the RN set by including the rest of the U that were predicted as "negative". In practice, the iteration was halted when no new negative samples were included or the number of iterations reached 20. Here, participants without HIV without PU Bagging Scores calculated were classified in the "unprotected" class by default.

Supervised Machine Learning

Random forest classifiers were trained with all humoral response features with stratified 5-fold cross-validation for hyperparameter tuning. Centering and Scaling were used to process the immune features that presented different ranges of values. Features were ranked according to impurity-based

feature importance under the label of inferred protection (i: protection) as candidate Correlates of Protection (CoPs) and under infection outcome as Correlates of Risk (CoRs). In this work, the top-ranked features with an importance score > 0.15 were shown. AUC and F1 scores under 5-fold cross-validation were employed to evaluate the generalizability of the model. In 100-time repeated modeling, the pooled importance score of selected features was compared between that under the acquisition outcome and inferred label of varied protection level. The model was implemented using the scikit-learn library (version 1.1.3) on Python 3.11 [46].

Statistical Analysis

Mann Whitney U tests were used to exclude the non-vaccine-elicited humoral responses from the feature set and test for the significance of immune features associated with infection or inferred protection status. Logistic regression models were built to assess the correlation between predicted outcome or clinical endpoint to immune features with covariates adjusted (age, BMI, race, behavioral risk score). The Benjamini-Hochberg procedure was performed to adjust for multiple hypothesis testing. A p-value of 0.05 was used as a cutoff to determine statistical significance. Mann Whitney U tests were performed using SciPy library on Python 3.11[47]; the Logistic regression model was implemented in R (version 4.2.2).

Study approval

Participants in HVTN 505 (NCT00865566) provided written informed consent [3] for both the main study and this additional use of their samples. Analysis of HVTN 505 samples reported here was reviewed and approved through the Institutional Review Board at Dartmouth College.

Data and Material Availability

The datasets used in this analysis are available at https://atlas.scharp.org/project/HVTN%20Public%20Data/HVTN%20505/begin.view.

Acknowledgements:

This study was supported in part by NIAID R56AI165448 and P01AI162242 and by the HVTN505 study team NIH 5UM1AI068618. This study was supported in part by the Dartmouth International Vaccine Initiative.

Disclaimer

The content is solely the responsibility of the authors and does not necessarily represent the official views of the National Institutes of Health.

Author Contributions:

The investigation, coding, data visualization, writing- original draft: S.X.; Writing- reviewing and editing: M.E.A. Supervision: M.E.A.; Conceptualization and funding: M.E.A.

References

1. Ng'uni, T., C. Chasara, and Z.M. Ndhlovu, *Major Scientific Hurdles in HIV Vaccine Development: Historical Perspective and Future Directions.* Front Immunol, 2020. **11**: p. 590780.
2. Rerks-Ngarm, S., et al., *Vaccination with ALVAC and AIDSVAX to Prevent HIV-1 Infection in Thailand.* New England Journal of Medicine, 2009. **361**(23): p. 2209-2220.
3. Hammer, S.M., et al., *Efficacy trial of a DNA/rAd5 HIV-1 preventive vaccine.* N Engl J Med, 2013. **369**(22): p. 2083-92.
4. Decamp, A.C., et al., *Sieve analysis of breakthrough HIV-1 sequences in HVTN 505 identifies vaccine pressure targeting the CD4 binding site of Env-gp120.* PloS one, 2017. **12**(11): p. e0185959.
5. Fong, Y., et al., *Modification of the association between T-cell immune responses and human immunodeficiency virus type 1 infection risk by vaccine-induced antibody responses in the HVTN 505 trial.* The Journal of infectious diseases, 2018. **217**(8): p. 1280-1288.
6. Neidich, S.D., et al., *Antibody Fc effector functions and IgG3 associate with decreased HIV-1 risk.* J Clin Invest, 2019. **129**(11): p. 4838-4849.
7. Corey, L., et al., *Two Randomized Trials of Neutralizing Antibodies to Prevent HIV-1 Acquisition.* N Engl J Med, 2021. **384**(11): p. 1003-1014.
8. Mkhize, N.N., et al., *Neutralization profiles of HIV-1 viruses from the VRC01 Antibody Mediated Prevention (AMP) trials.* PLoS Pathog, 2023. **19**(6): p. e1011469.
9. Seaton, K.E., et al., *Pharmacokinetic serum concentrations of VRC01 correlate with prevention of HIV-1 acquisition.* EBioMedicine, 2023. **93**: p. 104590.
10. Gilbert, P.B., et al., *Neutralization titer biomarker for antibody-mediated prevention of HIV-1 acquisition.* Nat Med, 2022. **28**(9): p. 1924-1932.
11. Reeves, D.B., et al., *High monoclonal neutralization titers reduced breakthrough HIV-1 viral loads in the Antibody Mediated Prevention trials.* Nat Commun, 2023. **14**(1): p. 8299.
12. Kelkar, N.S., K.S. Morrison, and M.E. Ackerman, *Foundations for improved vaccine correlate of risk analysis using positive-unlabeled learning.* Human Vaccines & Immunotherapeutics, 2023: p. 2204020.
13. S., X., N.S. Kelkar, and M.E. Ackerman, *Positive-unlabeled learning to infer protection status and identify correlates in vaccine efficacy field trials.* iScience, 2024. **27**(3).
14. Xu, S. and M.E. Ackerman, *Leveraging permutation testing to assess confidence in positive-unlabeled learning applied to high-dimensional biological datasets.* BMC Bioinformatics, 2024. **25**(1): p. 218.
15. Janes, H.E., et al., *Higher T-Cell Responses Induced by DNA/rAd5 HIV-1 Preventive Vaccine Are Associated With Lower HIV-1 Infection Risk in an Efficacy Trial.* J Infect Dis, 2017. **215**(9): p. 1376-1385.
16. Mordelet, F. and J.P. Vert, *A bagging SVM to learn from positive and unlabeled examples.* Pattern Recognition Letters, 2014. **37**: p. 201-209.
17. Liu, B., et al. *Partially supervised classification of text documents*. in *ICML*. 2002. Sydney, NSW.

18. Breiman, L., *Random forests.* Machine learning, 2001. **45**: p. 5-32.
19. Haynes, B.F., et al., *Immune-correlates analysis of an HIV-1 vaccine efficacy trial.* N Engl J Med, 2012. **366**(14): p. 1275-86.
20. Tomaras, G.D., et al., *Vaccine-induced plasma IgA specific for the C1 region of the HIV-1 envelope blocks binding and effector function of IgG.* Proc Natl Acad Sci U S A, 2013. **110**(22): p. 9019-24.
21. Gray, G.E., et al., *Vaccine efficacy of ALVAC-HIV and bivalent subtype C gp120–MF59 in adults.* New England Journal of Medicine, 2021. **384**(12): p. 1089-1100.
22. Johnson, J., *Johnson & Johnson and Global Partners Announce Results from Phase 2b Imbokodo HIV Vaccine Clinical Trial in Young Women in Sub-Saharan Africa*. 2021.
23. Johnson, J., *Janssen and Global Partners to Discontinue Phase 3 Mosaico HIV Vaccine Clinical Trial.* 2023.
24. Buchbinder, S.P., et al., *Efficacy assessment of a cell-mediated immunity HIV-1 vaccine (the Step Study): a double-blind, randomised, placebo-controlled, test-of-concept trial.* Lancet, 2008. **372**(9653): p. 1881-1893.
25. Gaiha, G.D., et al., *Structural topology defines protective CD8(+) T cell epitopes in the HIV proteome.* Science, 2019. **364**(6439): p. 480-484.
26. Leggat, D.J., et al., *Vaccination induces HIV broadly neutralizing antibody precursors in humans.* Science, 2022. **378**(6623): p. eadd6502.
27. Verweij, M.C., et al., *Modulation of MHC-E transport by viral decoy ligands is required for RhCMV/SIV vaccine efficacy.* Science, 2021. **372**(6541).
28. Saunders, K.O., et al., *Broadly Neutralizing Human Immunodeficiency Virus Type 1 Antibody Gene Transfer Protects Nonhuman Primates from Mucosal Simian-Human Immunodeficiency Virus Infection.* J Virol, 2015. **89**(16): p. 8334-45.
29. Ackerman, M.E., et al., *Route of immunization defines multiple mechanisms of vaccine-mediated protection against SIV.* Nat Med, 2018. **24**(10): p. 1590-1598.
30. Sanders, R.W., et al., *A next-generation cleaved, soluble HIV-1 Env trimer, BG505 SOSIP.664 gp140, expresses multiple epitopes for broadly neutralizing but not non-neutralizing antibodies.* PLoS Pathog, 2013. **9**(9): p. e1003618.
31. Chung, A.W., et al., *Polyfunctional Fc-effector profiles mediated by IgG subclass selection distinguish RV144 and VAX003 vaccines.* Sci Transl Med, 2014. **6**(228): p. 228ra38.
32. Chung, A.W., et al., *Dissecting Polyclonal Vaccine-Induced Humoral Immunity against HIV Using Systems Serology.* Cell, 2015. **163**(4): p. 988-98.
33. Yates, N.L., et al., *Vaccine-induced Env V1-V2 IgG3 correlates with lower HIV-1 infection risk and declines soon after vaccination.* Sci Transl Med, 2014. **6**(228): p. 228ra39.
34. Tomaras, G.D., et al., *Vaccine-induced plasma IgA specific for the C1 region of the HIV-1 envelope blocks binding and effector function of IgG.* Proceedings of the National Academy of Sciences, 2013. **110**(22): p. 9019-9024.
35. Fischinger, S., et al., *IgG3 collaborates with IgG1 and IgA to recruit effector function in RV144 vaccinees.* JCI insight, 2020. **5**(21).
36. Alter, G., et al., *Passive Transfer of Vaccine-Elicited Antibodies Protects against SIV in Rhesus Macaques.* Cell, 2020. **183**(1): p. 185-196 e14.
37. Barouch, D.H., et al., *Protective efficacy of adenovirus/protein vaccines against SIV challenges in rhesus monkeys.* Science, 2015. **349**(6245): p. 320-4.

38. Barouch, D.H., et al., *Evaluation of a mosaic HIV-1 vaccine in a multicentre, randomised, double-blind, placebo-controlled, phase 1/2a clinical trial (APPROACH) and in rhesus monkeys (NHP 13-19).* Lancet, 2018. **392**(10143): p. 232-243.
39. Shangguan, S., et al., *Monocyte-derived transcriptome signature indicates antibody-dependent cellular phagocytosis as a potential mechanism of vaccine-induced protection against HIV-1.* Elife, 2021. **10**.
40. Kenny, A., et al. *Immune correlates analysis of the Imbokodo HIV-1 vaccine efficacy trial*. in *AIDS 2022*. 2022. Montreal, CA.
41. Moodie, Z., et al., *Analysis of the HIV Vaccine Trials Network 702 Phase 2b-3 HIV-1 Vaccine Trial in South Africa Assessing RV144 Antibody and T-Cell Correlates of HIV-1 Acquisition Risk.* J Infect Dis, 2022. **226**(2): p. 246-257.
42. Ackerman, M.E., et al., *A robust, high-throughput assay to determine the phagocytic activity of clinical antibody samples.* J Immunol Methods, 2011. **366**(1-2): p. 8-19.
43. Tomaras, G.D., et al., *Initial B-cell responses to transmitted human immunodeficiency virus type 1: virion-binding immunoglobulin M (IgM) and IgG antibodies followed by plasma anti-gp41 antibodies with ineffective control of initial viremia.* J Virol, 2008. **82**(24): p. 12449-63.
44. Brown, E.P., et al., *Optimization and qualification of an Fc Array assay for assessments of antibodies against HIV-1/SIV.* J Immunol Methods, 2018. **455**: p. 24-33.
45. Brown, E.P., et al., *Multiplexed Fc array for evaluation of antigen-specific antibody effector profiles.* J Immunol Methods, 2017. **443**: p. 33-44.
46. Pedregosa, F., et al., *Scikit-learn: Machine Learning in Python.* Journal of Machine Learning Research, 2011. **12**: p. 2825-2830.
47. Virtanen, P., et al., *SciPy 1.0: fundamental algorithms for scientific computing in Python.* Nat Methods, 2020. **17**(3): p. 261-272.

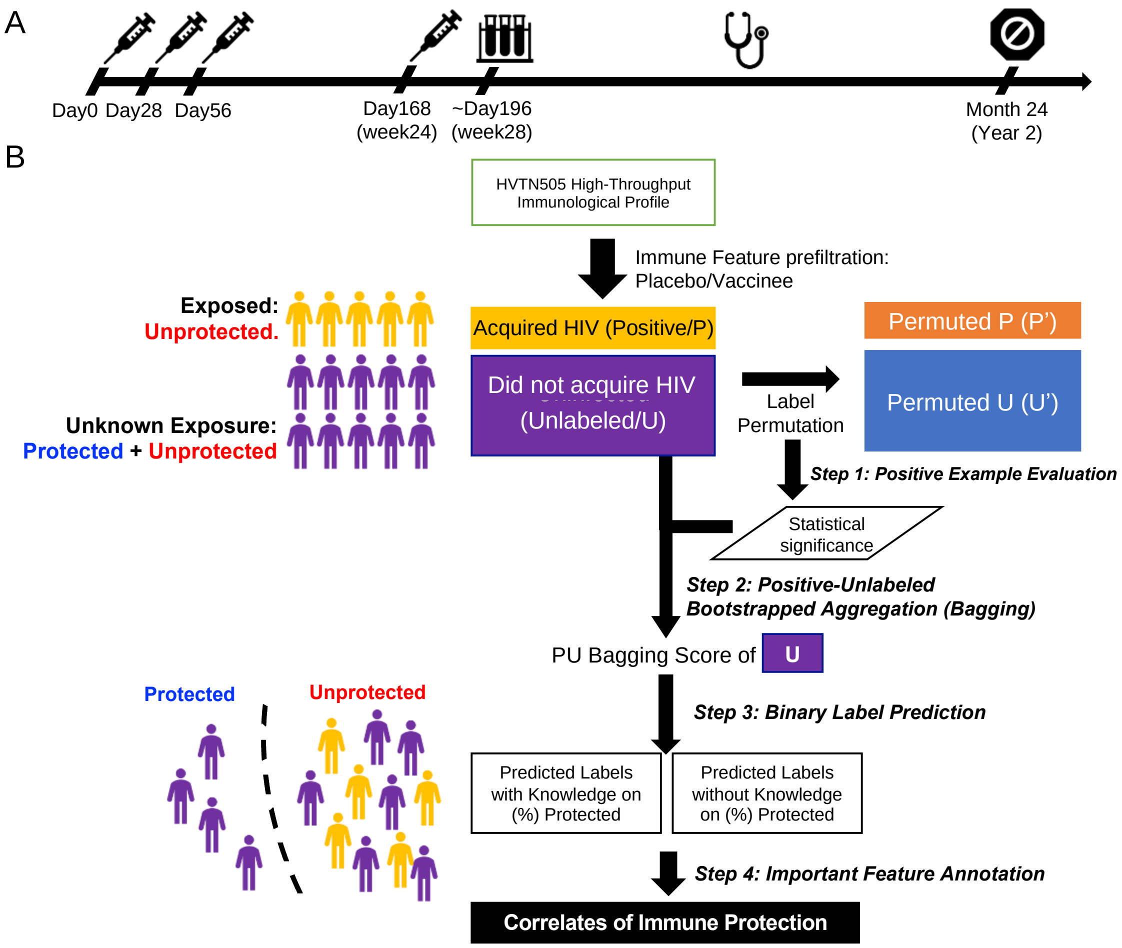

Figure 1: Positive-Unlabeled Learning-based pipeline to identify correlates of immune protection in HVTN505. A. Timeline of HVTN505 Trial. Study participants received placebo or a recombinant DNA plasmid vaccine injection on days 0, 28 and 56 and a recombinant adenoviral serotype vector vaccine injection on day 168. Immune responses to vaccination were assessed on week 28 and the clinical endpoint (infection) monitored between week 28 and month 24. **B**. Schematic of PU Learning approach to infer protection status. Vaccine recipients that have acquired HIV are definitively unprotected and represent known positives (P). Unlabeled (U), or vaccine recipients that have not acquired HIV are hypothesized to include both protected participants as well as unprotected participants who were simply not exposed. In Step 1, a PU Bagging classifier was used to define class probabilities for actual PU labels and permuted PU class labels. In Step 2, the PU Bagging scores for participants in the unlabeled (U) class were defined. In Step 3, these class scores were used to generate a binary protection status class inference. In Step 4, these inferred protection status class labels are used to discover new candidate correlates of protection.

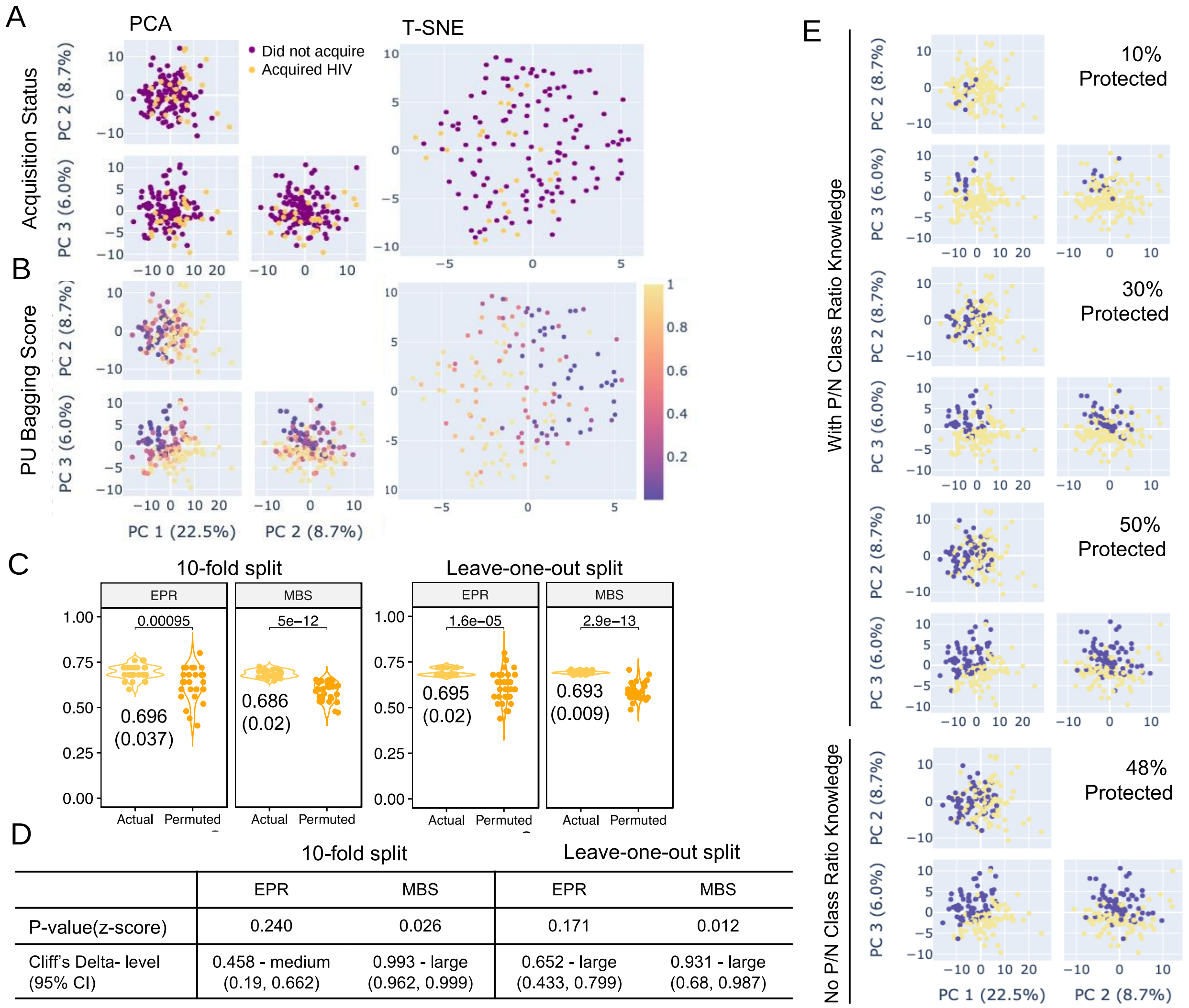

	10-fold split EPR	10-fold split MBS	Leave-one-out split EPR	Leave-one-out split MBS
P-value(z-score)	0.240	0.026	0.171	0.012
Cliff's Delta- level (95% CI)	0.458 - medium (0.19, 0.662)	0.993 - large (0.962, 0.999)	0.652 - large (0.433, 0.799)	0.931 - large (0.68, 0.987)

Figure 2: PU learning-based inference of protection status. A-B. Principal component analysis (PCA, left) and T-SNE (right) to visualize the global structure of the dataset. Samples are labeled according to the HIV acquisition status outcome (**A**) or inferred protection status score obtained from the PU Bagging algorithm (**B**). **C.** Explicit Positive Recall (EPR, left) and Mean Bagging Score (MBS, right) value distributions were calculated for known positive class (Acquired HIV) samples for models trained on actual (yellow) and permuted (orange) class labels. Mean and (standard deviation (SD)) of scores are presented below each distribution. **D**. Statistical analysis comparing class probability scores of known actual and permuted positive samples. Statistical significance between score distributions was defined by Two-sample independent T-test, p-value from z-score, and effect size estimated using Cliff's Delta. **E.** PC biplots depicting classification of unlabeled samples with knowledge of the ratio between protected and unprotected subjects among the unlabeled class set to 10, 30, and 50% (from top), or without the benefit of knowledge of the proportion of positive and negative classes anticipated among unlabeled samples (bottom).

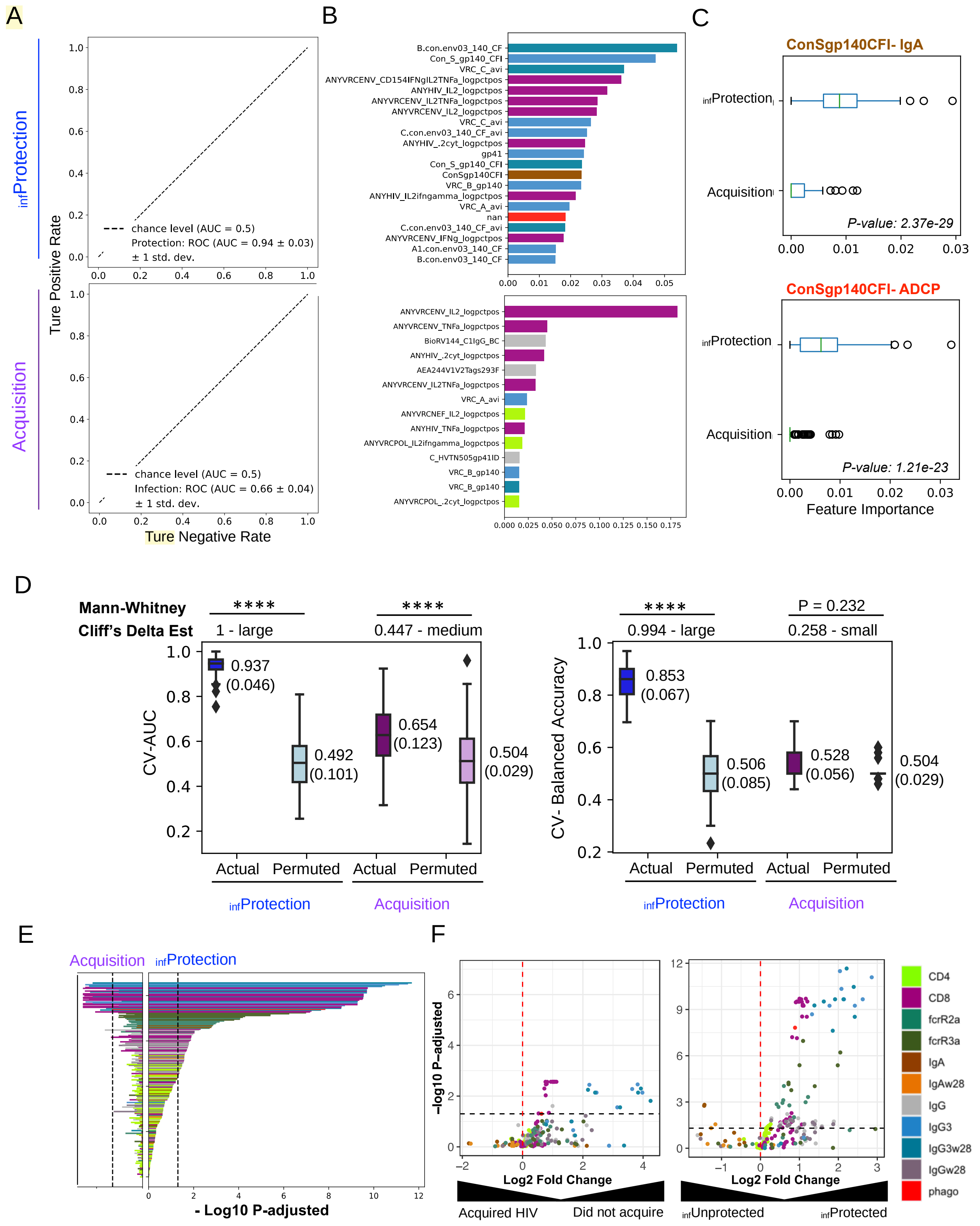

A
infProtection
Acquisition
Ture Positive Rate
Ture Negative Rate
chance level (AUC = 0.5)
Protection: ROC (AUC = 0.94 ± 0.03)
± 1 std. dev.
chance level (AUC = 0.5)
Infection: ROC (AUC = 0.66 ± 0.04)
± 1 std. dev.
B
B.con.env03_140_CF
Con_S_gp140_CFI
VRC_C_avi
ANYVRCENV_CD154IFNgIL2TNFa_logpctpos
ANYHIV_IL2_logpctpos
ANYVRCENV_IL2TNFa_logpctpos
ANYVRCENV_IL2_logpctpos
VRC_C_avi
C.con.env03_140_CF_avi
ANYHIV_.2cyt_logpctpos
gp41
Con_S_gp140_CFI
ConSgp140CFI
VRC_B_gp140
ANYHIV_IL2ifngamma_logpctpos
VRC_A_avi
nan
C.con.env03_140_CF_avi
ANYVRCENV_IFNg_logpctpos
A1.con.env03_140_CF
B.con.env03_140_CF
ANYVRCENV_IL2_logpctpos
ANYVRCENV_TNFa_logpctpos
BioRV144_C1IgG_BC
ANYHIV_.2cyt_logpctpos
AEA244V1V2Tags293F
ANYVRCENV_IL2TNFa_logpctpos
VRC_A_avi
ANYVRCNEF_IL2_logpctpos
ANYHIV_TNFa_logpctpos
ANYVRCPOL_IL2ifngamma_logpctpos
C_HVTN505gp41ID
VRC_B_gp140
VRC_B_gp140
ANYVRCPOL_.2cyt_logpctpos
C
ConSgp140CFI- IgA
infProtection
Acquisition
P-value: 2.37e-29
ConSgp140CFI- ADCP
infProtection
Acquisition
P-value: 1.21e-23
Feature Importance
D
Mann-Whitney
Cliff's Delta Est
****
****
1 - large
0.447 - medium
CV-AUC
0.937 (0.046)
0.492 (0.101)
0.654 (0.123)
0.504 (0.029)
Actual
Permuted
Actual
Permuted
infProtection
Acquisition
****
P = 0.232
0.994 - large
0.258 - small
CV- Balanced Accuracy
0.853 (0.067)
0.506 (0.085)
0.528 (0.056)
0.504 (0.029)
Actual
Permuted
Actual
Permuted
infProtection
Acquisition
E
Acquisition
infProtection
- Log10 P-adjusted
F
–log10 P–adjusted
Log2 Fold Change
Acquired HIV
Did not acquire
Log2 Fold Change
infUnprotected
infProtected
CD4
CD8
fcrR2a
fcrR3a
IgA
IgAw28
IgG
IgG3
IgG3w28
IgGw28
phago

Figure 3: Classification and correlates under inferred protection as compared to infection status. A. Receiver Operator Characteristic (ROC) curves of a Random Forest classification model under stratified 5-fold cross validation to predict inferred protection ($_{inf}$Protection, top, in blue) or acquisition (bottom, in purple) status. Gray shaded area represents ±1 SD of the performance in each fold. Dotted line indicates the performance expected at random, and area under the curve (AUC) values are reported in inset. **B.** Feature importance scores of the top ranked humoral response features, colored by feature type, for each classification task. **C.** Boxplots depicting feature importance scores for Con S Env gp140-specific IgA and ADCP for inferred protection and acquisition class labels in 100-time repeated modeling. Statistical significance was defined by Mann-Whitney U test. **D.** Boxplots of model performance assessed by permutation test. 30-time repeated cross-validated AUC (left) and Balanced Accuracy (right) under predicted protection level and acquisition label was compared to that under randomly shuffled labels for each (permuted labels). The multiple-time cross-validated performance was annotated with the format in Mean (Standard Deviation). Mann Whitney U test and Cliff's Delta Estimate (annotated on the top of the panels) were employed to determine the statistical significance between groups (****: p-value < 0.0001). **(E-F)** Identification of correlates. **E**. Mann-Whitney U tests were employed to compare each immunogenicity response feature for HIV acquisition status (left) and inferred protection status (right) class labels. Features are ordered on y-axis according to the rank of -Log10 p-adjusted obtained from protection label. **F.** Volcano plots depicting confidence and magnitude of differences in immune response features associated with HIV acquisition (left) and inferred protection (right) status. The Benjamini-Hochberg procedure was used to adjust p-values from Mann Whitney U test to reduce the false discovery rate.

A

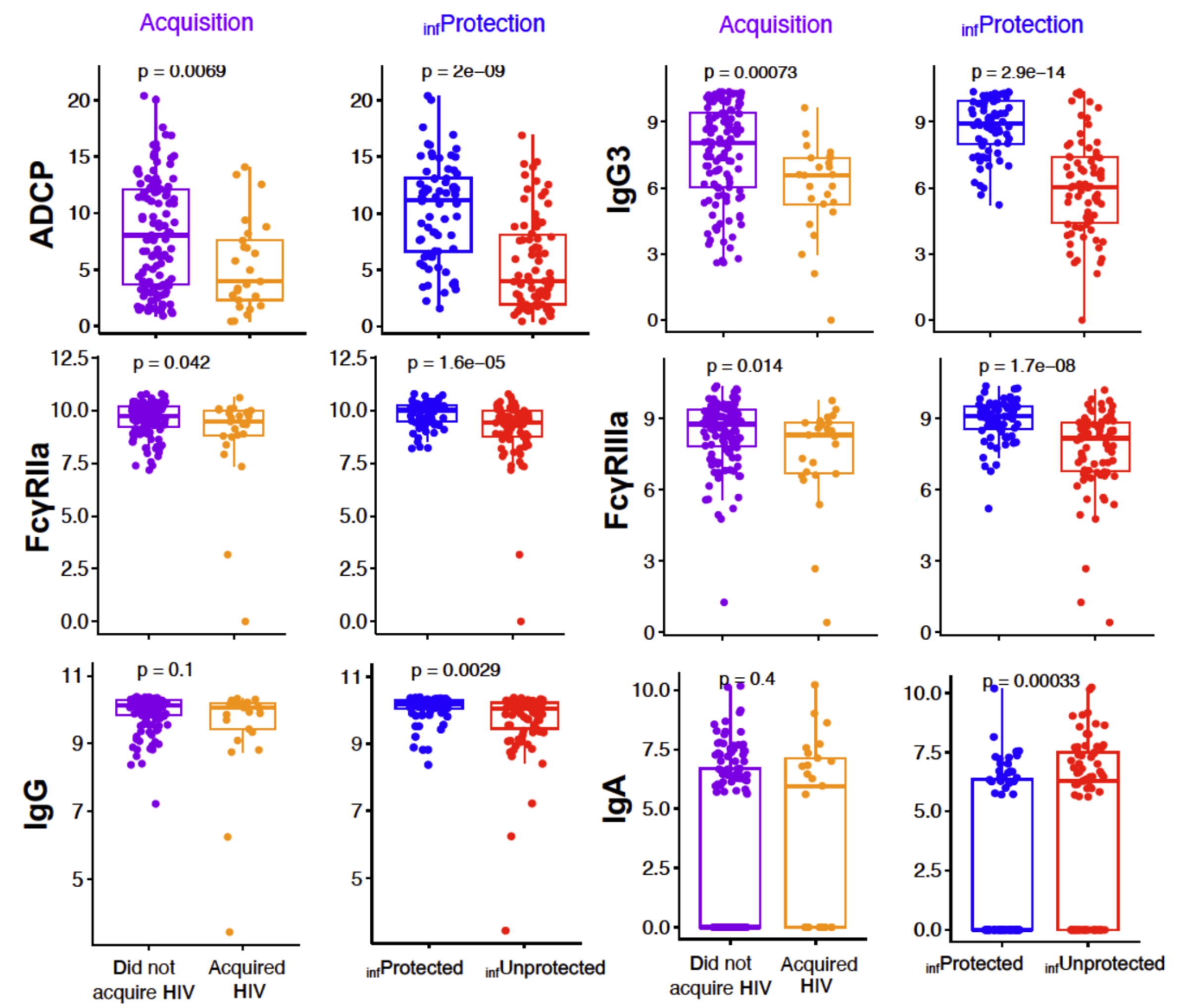

B

	Mann Whitney U test				Logistic Regression Model			
	P-unadjusted		P-adjusted		P-unadjusted		P-adjusted	
	Acquisition	$_{inf}$Protection	Acquisition	$_{inf}$Protection	Acquisition	$_{inf}$Protection	Acquisition	$_{inf}$Protection
**ADCP**	0.00692	2.04e-09	0.0498	1.52e-08	0.00675	1.16e-07	0.0430	9.21e-07
**IgG3**	0.000728	2.90e-14	0.00732	3.24e-12	0.000905	7.99e-10	0.0151	9.94e-08
**FcγRIIa**	0.0421	1.64e-05	0.190	**9.14e-05**	0.00601	3.06e-05	0.0406	0.000171
**FcγRIIIa**	0.0141	1.66e-08	0.0950	**1.09e-07**	0.00498	1.26e-06	0.0370	8.00e-06
**IgG**	0.101	**0.00294**	0.322	**0.0113**	0.0160	0.00170	0.0774	**0.00620**
**IgA**	0.403	**0.000327**	0.628	**0.00152**	0.576	**0.00120**	0.742	**0.00462**

Figure 4: Comparisons of ConS gp140-specific features by infection and inferred protection status labels. A. Boxplots of raw value distribution of notable ConS gp140 Env-specific humoral response measurements according to HIV acquisition and inferred protection ($_{inf}$Protection) status classes. Statistical significance defined by T test. **B.** Table of p values from Mann Whitney U test and Logistic regression model following adjustments based on clinical traits as covariates. P-unadjusted: p-values directly obtained from bivariate tests (left) or logistic regression models (right). P-adjusted: Benjamini-Hochberg method was used to reduce false discovery rate for multiple test hypothesis. Newly inferred candidate correlates are indicated in bold.

Click here to access/download
Supporting Information
Supplemental Xu HVTN505.pdf
