# Supplementary material for: Candidate correlates of protection in the HVTN505 HIV-1 vaccine efficacy trial identified by positive-unlabeled learning
Source: PLoS Comput Biol. 2025 Nov 26;21(11):e1013705. doi: 10.1371/journal.pcbi.1013705 (PMC12654939; doi:10.1371/journal.pcbi.1013705)
Supplement: Attachment — Submitted filename: response to review_R1.pdf [file pcbi.1013705.s003.pdf]

Reviewers' comments:

Reviewer's Responses to Questions

Comments to the Authors:
Please note that one of the reviews is uploaded as an attachment.

Reviewer #1: In the present article, Xu et al. employ a Positive-Unlabeled (PU) inference method on immunogenicity data collected in the HVTN 505 study to infer vaccine-mediated protection scores against HIV acquisition by comparing patient immunogenicity profiles with those who acquired HIV. The authors then regressed these protection scores against the immunogenicity data to identify correlates of protection (CoP) against HIV infection. To test the robustness of their scores, the authors employed permutation testing, finding that their protection scores and CoPs were not artifacts of chance. These efforts confirmed previously reported correlates of risk (CoR) as well as identified novel CoPs, such as Env gp140-specific IgA and ADCP, that were not found through HIV acquisition-based models. Overall, this study is a well-reasoned application of novel machine learning methods to improve recognition of biological determinants in PU data that may also progress our understanding of CoPs in HIV vaccination. However, further efforts to verify the biological validity of the protection scores would greatly bolster confidence in PU inference methods and in the CoPs identified in this article. There are also several opportunities to improve the clarity of the figures and text.

Major Concerns

Figure 3A is missing the prediction lines.

Thank you, this visualization has been checked to display appropriately in the uploaded TIFF file.

It is claimed that there is some clustering in Figure 2A among positive samples, but is this really supported? Adding quantification of clustering separation, such as through the Silhouette score, would improve confidence in this claim.

We have substituted the word “similarity” for “clustering” in describing this figure, as we agree that “clustering” suggested a degree of separation between positive and unlabeled groups that is not observed.

The authors use permutation testing to validate their Positive-Unlabeled (PU) model captured immunological patterns different from those in randomized measurements. However, it remains unclear if the patients identified as protected from HIV infection were truly protected from infection or if they were never exposed to HIV. To their credit, the authors acknowledge that their permutation efforts cannot verify if the inferred protection statuses are biologically correct, and that such verification is not possible as patient HIV exposures are unknown. In the supplement, the authors conclude there is no significant association between behavioral vulnerability and inferred protection status, but that there is an association between behavioral vulnerability and HIV acquisition. As such, behavioral vulnerability could be used as a proxy for HIV exposure. Perhaps the biological validity of the PU-inferred protection may be bolstered by comparing inferred protection between acquisition and non-acquisition cases in patients with high behavioral vulnerability.

Thank you for this excellent suggestion. Scores were generated for participants with high behavioral vulnerability, whether in the P or U class. P class participant scores were calculated when they were unlabeled and included as spy positives in the setting of leave one out cross-validation.

When comparing PU score among all high behavioral risk participants, we do indeed see some evidence supporting the validity of our protection status inferences (**Response to revision Figure 1, Revised manuscript Figure 2**).

High behavioral risk participants who do not acquire HIV present lower PU scores (greater probability of being protected) than do those that did acquire HIV (Welch's Test, p=0.047). The median PU score calculated for uninfected participants with high behavior risk was approximately 0.5, whereas the median PU score of high risk participants that did acquire HIV was 0.8.

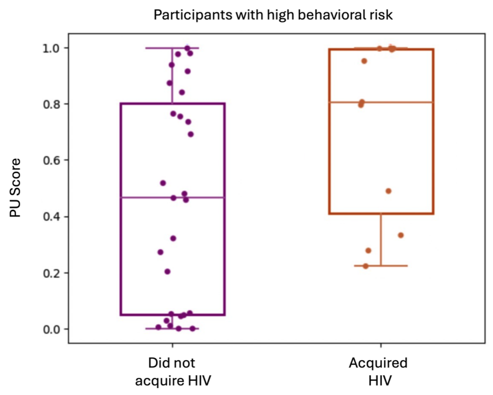

Response to revision Figure 1. Support for protection status inference validity. PU score for high behavioral risk participants who did not acquire HIV (blue), and those who did (red). Welch's test p=0.047.

Minor Concerns

In line 68, the statement "While over-representation of controls is a commonly used means to improve upon the ability to detect CoR…" should include a citation to support its claim that oversampling is a common method in CoR.

We have added references.

In line 148, missing a "were" between "that" and "observed".

corrected

Line 164: The authors allude to two-sample independent t-tests in Figure 2D, though the table in Figure 2D does not include t-test results. Similarly, it is unclear which statistical test was used to define significance differences in EPR and MBS means for Figure 2C.

We apologize for lack of clarity here. The legend has been revised to specify that T test results are shown in the inset of Figure 2C rather than tabulated in Figure 2D.

The color for the lighter colored data points, such as yellow, should be adjusted so they can be seen more easily.

Panel E (the ones with very light yellow) in figure 2 was changed into blue/red matched to what we have in Figure 4. Color schemes in panel A, B and C remained same because these colors were repeatedly mapped to that used in other figures.

Figures 2A-B should be reorganized for improved clarity. Reorganizing into two columns, with each row representing a unique combination of principal components and each column corresponding to HIV acquisition or inferred protection, may better illustrate relationships between acquisition and inferred protection.

Figures 2A-B tiling has been revised as suggested.

Figure 2B contains “nan” as a feature of importance. Is this a real feature? Can the authors include a dictionary of the features?

Figure 2B has been corrected to include clearer feature names that follow the more explanatory labels provided in the linked study data’s data dictionary files.

The triangles along the x-axis in Figure 3F should have their intersection at 0 log fold change, not the center of the plot.

This positioning has been corrected.

In Figure 3B, the x-axis should be labeled, and a legend should be included to define what types of features each color represents; if these correspond to the legend in 3F, it would be beneficial to put the legend earlier with Figure 3B. Similarly, it is unclear which classification task each feature importance plot corresponds to. Presumably, these match the classification task labels in Figure 3A, but increasing the spacing between rows or adding some background shading across all figures corresponding to a classification task may improve clarity in this distinction.

The suggested labeling edits have been included.

Throughout Figure 4, the authors should report Mann-Whitney U test statistics alongside p-values when comparing acquisition and protection models; p-values should be used to define if a difference in means is significant, whereas the test statistics should be used to compare deviations in means across models.

We do not compare acquisition and protection models in Figure 4, and so are not certain as to how to properly address this comment. However, for the Mann-Whitney tests of relationships between selected immunogenicity features and acquisition and inferred protection status, we have added U test statistics as **Supplemental Table 2**.

Reviewer #2: This study analyzes data from HVTN 505, a Phase IIb HIV vaccine trial that failed to meet its efficacy criteria, using a novel machine learning approach called Positive-Unlabeled (PU) learning to gain new insights about vaccine-mediated protection. While the trial showed no overall efficacy, previous analyses suggested that some vaccine recipients may have been protected from certain viral strains. The researchers applied PU learning to infer protection status among vaccine recipients who didn't acquire HIV, allowing for improved detection of potential correlates of immunity. Using this approach, the study confirmed previously identified correlates of risk, such as vaccine-elicited anti-HIV-1 Env glycoprotein IgG3 antibodies and antibody-dependent phagocytosis, while also revealing new observations including a strong inverse correlation between vaccine-mediated protection and virus-specific IgA responses. The findings demonstrate the value of advanced analytical methods for extracting meaningful insights from failed vaccine trials, particularly in cases where traditional analysis methods may lack statistical power due to low efficacy and exposure rates. This analytical framework offers a new way to use case-control datasets to identify markers of effective immune responses even in the context of low overall vaccine efficacy.

Major Comments:

1. Circular Logic and Validation Concerns

- The paper uses the same immunological data both to infer protection status and then to identify correlates of protection, creating problematic circular logic.
- The validation approach using permutation testing is insufficient to prove the biological relevance of the inferred protection classifications.

We agree that use of the same data for class inferences as well as for correlate testing, and the inability to demonstrate the biological relevance of the inferred protection classifications are fundamental limitations. We have revised the manuscript from abstract through discussion to better call attention to these limitations. (e.g. abstract: "**analytical** robustness"; "**though its biological validity is not established**"; "**candidate** markers of effective immune responses")

2. Statistical and Methodological Issues
- The PU learning approach makes strong assumptions about the mixture of protected/unprotected individuals that are not well justified.

Assumptions made by the PU learning algorithm are now listed in the discussion and their reasonableness is now specifically discussed and justified.

- Multiple testing corrections appear inadequate given the large number of features examined.

We are unsure of the basis for this statement, so we may not be able to formulate a very specific response. Nonetheless, while no correction method is perfect, we applied Benjamini-Hochberg Procedure MHC to control for false positives when multiple immunogenicity features were compared between either acquisition or inferred protection status groups (e.g. **Figure 3E,F, Figure 4, Supplemental Figure 3**).

Additionally, the revision has added language about appropriate caution in interpretation, considering both statistical significance and effect sizes. We welcome any suggestions on specific methods or components of the analysis that may better address this comment.

- The robustness of the protection status inferences across different modeling choices is not thoroughly evaluated.

Different modeling choices have been evaluated in prior methods studies and were therefore intentionally not varied here.

Here, a consensus scoring metric derived from different bagging folds and replicates was used to incorporate robustness and reproducibility of protection status inferences as alternative to making different modeling choices. To address the reproducibility of inferences as a means of characterizing model robustness, we include additional data in the revised manuscript. We find that class scores for the 125 unlabeled samples show the following variance across 30 modeling iterations (repeated scoring under different bagging seeds). This result has been added as **Response to Revision Figure**

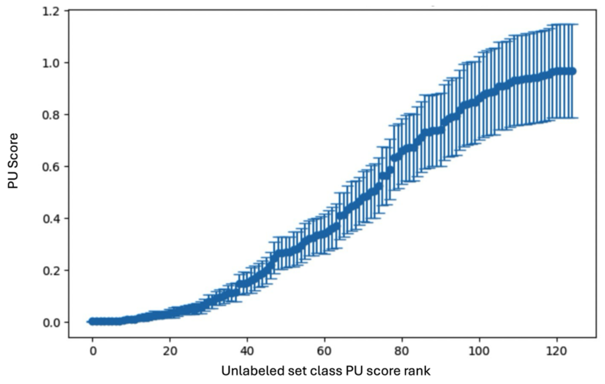

Response to revision Figure 2. Score robustness. PU scores for the unlabeled class across modeling iterations (repeated scoring under different bagging seeds). Symbol indicates mean and error bars indicate standard deviation. Samples are ordered from smallest to largest PU score.

2 and Revised manuscript Supplemental Figure 1.

- Inconsistent choice of k-fold from 3 (SVM), 5 (RF), to 10 (Bagging)!

Because these 3 methods were not used for the same task (and are not compared), it was not felt necessary to be consistent.

- Inconsistent use of softwares, Mann Whitney U tests were performed using SciPy library on Python 3.11[47]; the Logistic regression model was implemented in R (version 4.2.2). Why not using Wilcoxon test in R instead of Mann Whitney U test in SciPy? Or if Python is a preferred programming language, why not using SciKit Learn to perform Logistic Regression?

The statistical test results were generated alongside the figure generation in either R or python. The authors do not have preference of one language or package over the other to generate statistical significance and results are expected to be relatively insensitive to this choice.

3. Overstatement of Findings
- Claims about "discovering" new correlates of protection are overstated given the circular nature of the analysis.

We have taken care to add words such as “**candidate**” or “**potential**” to appropriately temper descriptions of correlates discovered from inferring protection status.

- The biological plausibility and mechanistic relevance of the identified features is not adequately discussed.

Mechanistic connections between IgG3, phagocytosis, FcgRIIa-binding, and IgA are now more fully described and supported through references in the discussion section.

- The limitations of inferring protection status without exposure data are downplayed.

This limitation is now better specifically called out in the discussion.

4. Structural and Presentation Issues
- The methods section lacks sufficient detail about key modeling choices and parameters.

Code is described and provided. Choices made were based on our published prior work with this method and the sample sizes available in this data set.

- Figures are not well presented and do not effectively communicate the key findings.

In addition to changes noted above, the figures have been retitled to better communicate key findings.

- The discussion does not adequately address alternative interpretations of the results.

Alternative interpretations are now included in the discussion.

- The practical utility for vaccine development is unclear given the methodological limitations

We agree that further validation is needed to establish the biological or regulatory relevance of candidate correlates of protection and note this limitation in the final sentence of the discussion.

Additional Points:
- The manuscript requires significant editing for clarity and conciseness.
- Key controls and sensitivity analyses are missing.
- The statistical significance of many findings appears marginal.
- Important caveats and limitations are buried in the discussion.

We have attempted to improve the manuscript in each of these dimensions based on both these broad comments as well as the specific comments related to these comments detailed above. We would be happy to follow up to address specific advice.

The core idea of using machine learning to gain additional insights from vaccine trial data is interesting, but the current manuscript has fundamental flaws in its approach and overreaches in its conclusions. To be suitable for publication, the authors would need to:

1. Develop independent validation approaches that don't rely on circular logic.

There seem to be two levels at which the work could be validated – acquisition status prediction, and candidate correlate identification. We are unsure to which the reviewer refers and will do our best to address both.

For model predictions of inferred protection status, we both employ validation approaches standard for semi-supervised ML studies, such as the spy technique and repeated cross-validation, and we report evaluation metrics used in previously semi-supervised ML studies. Beyond this, our permutation testing strategy is a novel contribution to this space and establishes the robustness (favorable deviation from results expected at random) of learned models. **The ability to accurately predict HIV acquisition status when positive but not randomly drawn samples are <u>un</u>labeled is one form of validation of the class assignments made by the model.**

It is difficult to conceive of an ethical and feasible means of further validation for the model's class predictions. For example, validation of class predictions of participants that are unlabeled in this data set would require intentional exposure of human beings to HIV, which is obviously not possible. Completely independent replication would require the trial to be repeated, which is also infeasible. Lastly, testing this model on another HIV vaccine cohort is precluded by the lack of corresponding immunogenicity data for the few strategies that have been tested, and would also rest on the presumption that different vaccines would exhibit the same relationships between acquisition risk and immune responses induced.

As for independent validation of our "inferred correlates of protection", we suggest to the reviewer that this analysis aims to provide insight into how features contribute to model predictions rather than standing alone as predictions to be validated. We have worked to revise the manuscript to emphasize this point.

Here, too, independent validation seems an unreasonable standard, as another clinical efficacy trial of this vaccine concept would be required. Given the vaccine's poor efficacy, this too would likely be considered unethical. Thus, the closest we are able to come to providing independent support for the validity of our inferred correlates is our demonstration that our method: 1) identified inferred correlates not apparent from standard evaluation of the HVTN505 regimen, but that are correlates of protection observed in the RV144 regimen, and, 2) based on the analysis suggested by Reviewer 1, that within the high behavioral risk group, inferred protection (PU score) is associated with HIV acquisition (**Response to revision Figure 1, Revised manuscript Supplementary Figure 2**).

We are careful to describe our observations as inferences of protection status and inferred or candidate correlates of protection, and we propose that further independent validation is not required to support this language. In closing, we emphasize the PU Learning is not a conventional supervised

classifier and in some ways functions more analogously to unsupervised methods such as clustering (but made more rigorous via use of repeated cross-validation).

2. More thoroughly evaluate the robustness of their findings.

We perform repeated cross-validated analysis and permutation testing and have added reproducibility of PU scores across repeated modeling in revision.

3. Provide more rigorous statistical analyses.

We have used standard statistical approaches, presented p values and effect sizes, with adjustment for clinical covariates and multiple hypothesis testing. Additionally, permutation tests serve as an analytical negative control.

4. Substantially improve the clarity of presentation.

We have worked to make the presentation clear and accessible, and would be happy to further improve the clarity of the manuscript if specific points at which the presentation is unclear are identified.

Reviewer #3: See the attachment

Have the authors made all data and (if applicable) computational code underlying the findings in their manuscript fully available?

Reviewer #1: **No:** I didn't see the raw data or a dictionary of the variable names. It's possible I just missed this.

Reviewer #2: **No:** Only the datasets used in this analysis are available at https://atlas.scharp.org/project/HVTN%20Public%20Data/HVTN%20505/begin.view.

A data dictionary is available at this link.

Reviewer #3: None

This is a well-written and potentially useful work. It applies a modification of the author's previously published work on PU methods for CoR and CoP finding. The results suggest immunological measures to be considered in future HIV vaccine development.

A number of aspects of the article require substantial clarification. Also, several of the results used to suggest strength of the methods appear to be expected from self-consistency alone.

We have taken care to ensure the points of expected self-consistency are made more clear.

Authors did not answer disclosure question.

Disclosure statement has been added.

{Funding} information not provided

Funding statement has been added.

Line 40 – citation error

This citation has been corrected.

Line 69 - Please clarify how biasing sampling is not an example of over-representation of controls? The "while" seems out of place.

This sentence has been revised for clarity as we agree that biased sampling is such an example.

Line 107 - This number of cases (and controls) has previously been demonstrated to be more than adequate for standard statistical methods to identify sensitively and specifically. Includes methods such as logistic regression and time-to-event analyses in publications spanning at least 2021-2024. R packages and similar availability for those methods suggest the authors should easily be able to compare their results to those of prior work as a way of demonstrating that this is a novel capability.

We agree and better emphasize prior studies of HVTN505 using such approaches in the introduction and discussion in order to provide comparison and better convey the consistency observed between approaches. We have also eliminated the word "sparse" from the description of the 25 acquisition cases.

Line 109 - in introduction, the authors do not cite extensive literature on CoP and CoR techniques for highly effective vaccines and for diseases with low incidence rates, that, as here, result in relatively few cases. This omission could be perceived by some as bias towards their preferred approaches. Previous comment on comparison of methods applies here as well.

We have worked to round out citations in the introduction, but now also explicitly narrow the focus of this work to vaccines with low rather than high efficacy (like HVTN505). Our prior work with synthetic data (PMID 37133899), though not extensive, suggested that PU learning may not provide a great deal of value in the context of high vaccine efficacy.

Line 162 - The authors should define statistical significance criterion used. Also, it is typically recommended that this nomenclature be avoided if the test (criterion and its cutoff value) was not proposed before data were generated, so the authors should also indicate at what point in process the criterion was created.

We added description of the criterion, cutoff, and time at which these were established to the "statistical analysis" section of the methods and amended the language in line 162.

Line 175 – abbreviation not defined

This abbreviation is now spelled out.

Line 206 - It is not clear which processes were repeated in this analysis.

This sentence has been revised for clarity.

Line 207 - As the authors suggested about the RF results above, this might just be another "circular" or just self-consistent result of using the same data set to do the inference and to calculate importance scores of the inferences derived from that data.

We have amended the description of this result.

Line 223 - See previous comments - this would be expected as self-consistency given the features were used to generate inferred protection status.

We have likewise tempered this description as expected.

Figure 3 legend - a guide to the meaning of feature names would enable better interpretation of results. Also, is the legend for color code the same as that in lower right corner near 3F? Further, some colors will be difficult to distinguish for many readers: a combination of color and pattern (in bars) or shape (in dots) would likely be helpful. Authors should probably clarify.

We now include more easily interpretable text labels for the features in Figure 3B. We also more explicitly note the placement of the legend for color is in the bottom right of the figure.
